# A Case of Glossitis

**Published:** 1853-11

**Authors:** J. S. Weatherly


### *A Case of Glossitis.*

BY J. S. WEATHERLY, M. D.

Miss P., aged about fourteen years, rather slender, and of a nervo-lymphatic temperament, had been sick three days when I saw her, on the 8th of April last. The attack occurred after exposure by going about one afternoon with her shoes and stockings off. She complained first of sore throat with occasional chilliness, and the throat grew worse until the 7th, when she had considerable fever and her tongue began to swell and be painful. On the morning of the 8th, the fever being high, headache intense and swallowing difficult, my presence was requested, and I reached the patient at 8 o'clock, P. M. I found her with intense cephalagia, great difficulty of deglutition, even of liquids; tongue much swollen, protruding between the teeth, very painful and covered with whitish mucus. She spoke with great difficulty and only in a whisper; skin tolerably hot; pulse 145; bowels constipated; urine very red.

Prescribed comp. cathartic powder—a teaspoonful of sp. æther. nit. —¼ gr. sulph. morph.—and 7 drops tr. ver. viride, in a wineglassful of water, every three hours; also warm applications to the throat and a hot foot-bath.

April 8th, A. M. Tongue more swollen, and protruding far between the teeth; she has some cough; discharges a tough mucus from the mouth, head still aching; pulse 120—has been nauseated by the ver. viride; bowels have not acted. Applied a blister to the throat; attempted to get some salts, tinct. rhei., and aloes down, but failed, as every attempt at swallowing strangled her.

Seeing that something had to be done which would give at least temporary relief, I determined to scarify the tongue. The blade of a straight bistoury was placed flat upon the dorsum of the tongue, and being guided by the left fore-finger, was pushed as far down as it could be done conveniently; it was then turned upon the edge, and brought out. making a deep incision into the substance of the tongue. Two other incisions were made in the same manner, which bled freely.

12 o'clock M. Can talk distinctly, and is able to swallow. She now took the salts, rhei. and aloes—the blister was ordered to be removed and followed by a warm poultice—the ver. vir. and spts. of nitre to be continued—cathartic mixture to be repeated, if necessary.

Apil 10th, A. M. Patient nearly well. Bowels had acted freely; tongue reduced to nearly its original size; pulse 75; complains of nothing except the blister. No unfavorable symptoms occurred afterwards. She had a rapid canvalescence.

